# Understanding subcortical projections to the lateral posterior thalamic nucleus and its subregions using retrograde neural tracing

**DOI:** 10.3389/fnana.2024.1430636

**Published:** 2024-08-07

**Authors:** Hisashi Nakamura, Keisuke Ohta

**Affiliations:** ^1^Division of Microscopic and Developmental Anatomy, Department of Anatomy, Kurume University School of Medicine, Kurume, Japan; ^2^Advanced Imaging Research Center, Kurume University School of Medicine, Kurume, Japan

**Keywords:** lateral posterior nucleus, superior colliculus, fluorogold, pulvinar, periaqueductal gray, pretectal area

## Abstract

The rat lateral posterior thalamic nucleus (LP) is composed of the rostromedial (LPrm), lateral (LPl), and caudomedial parts, with LPrm and LPl being areas involved in information processing within the visual cortex. Nevertheless, the specific differences in the subcortical projections to the LPrm and LPl remain elusive. In this study, we aimed to reveal the subcortical regions that project axon fibers to the LPl and LPrm using a retrograde neural tracer, Fluorogold (FG). After FG injection into the LPrm or LPl, the area was visualized immunohistochemically. Retrogradely labeled neurons from the LPrm were distributed in the retina and the region from the diencephalon to the medulla oblongata. Diencephalic labeling was found in the reticular thalamic nucleus (Rt), zona incerta (ZI), ventral lateral geniculate nucleus (LGv), intergeniculate leaflet (IGL), and hypothalamus. In the midbrain, prominent labeling was found in the periaqueductal gray (PAG) and deep layers of the superior colliculus. Additionally, retrograde labeling was observed in the cerebellar and trigeminal nuclei. When injected into the LPl, several cell bodies were labeled in the visual-related regions, including the retina, LGv, IGL, and olivary pretectal nucleus (OPT), as well as in the Rt and anterior pretectal nucleus (APT). Less labeling was found in the cerebellum and medulla oblongata. When the number of retrogradely labeled neurons from the LPrm or LPl was compared as a percentage of total subcortical labeling, a larger percentage of subcortical inputs to the LPl included projections from the APT, OPT, and Rt, whereas a large proportion of subcortical inputs to the LPrm originated from the ZI, reticular formation, and PAG. These results suggest that LPrm not only has visual but also multiple sensory-and motor-related functions, whereas the LPl takes part in a more visual-specific role. This study enhances our understanding of subcortical neural circuits in the thalamus and may contribute to our exploration of the mechanisms and disorders related to sensory perception and sensory-motor integration.

## 1 Introduction

The lateral posterior thalamic nucleus (LP) in rodents is the primate equivalent of the pulvinar nuclei and is a component of the extrageniculate pathway that relays visual information from the retina and superior colliculus (SC) to the visual cortex (Diamond and Hall, 1969; Schneider, 1969; Jones, 2007; Tohmi et al., 2014). The pulvinar/LP is known to bidirectionally connect with several cortical areas, primarily the visual-related cortex (Kaas and Lyon, 2007; Bennett et al., 2019; Juavinett et al., 2020; Scholl et al., 2021). Additionally, these nuclei are associated with visual discrimination tasks and spatial attention (Hughes, 1977; Wilke et al., 2010). However, the pulvinar/LP is not a homogeneous structure but consists of multiple subnuclei, each forming distinct cortico-thalamic neural networks (Kaas and Lyon, 2007; Bennett et al., 2019). In addition, various subcortical inputs to the different intralaminar thalamic nuclei have recently been identified in the human brain (Kumar et al., 2023), indicating that there are different subcortically derived thalamic projections for each small area within the thalamus.

The LP in rats is divided into three subregions: caudomedial (LPcm), rostromedial (LPrm), and lateral (LPl) (Takahashi, 1985). The LPcm primarily receives input from the superficial layers of the SC (SC-s) and mainly projects to the posterior temporal cortex (Perry, 1980; Mason and Groos, 1981; Takahashi, 1985; Shi and Davis, 2001; Masterson et al., 2009; Nakamura et al., 2015; Baldwin et al., 2017; Zhou et al., 2017). In contrast, both the LPrm and LPl maintain strong reciprocal connections with the primary and secondary visual cortices and participate in cortical visual processing (Perry, 1980; Mason and Groos, 1981; Takahashi, 1985; Bourassa and Deschênes, 1995; Shi and Davis, 2001; Masterson et al., 2009; Blot et al., 2021). Furthermore, recent studies have also revealed differences in the cortical projections from the LPl and LPrm, as well as variations in the cortical inputs to these two nuclei (Nakamura et al., 2015; Bennett et al., 2019; Juavinett et al., 2020; Scholl et al., 2021). However, the specific roles of these two subnuclei remain poorly understood.

Reportedly, the LPl and LPrm receive inputs from subcortical regions other than the retina and SC (Power et al., 1999; Kolmac et al., 2000; Moore et al., 2000; Scholl et al., 2021; Leow et al., 2022). Notably, vesicular glutamate transporter 2, a marker for subcortical excitatory input, has been identified in high abundance within the LP nucleus (Kaneko et al., 2002). However, the specific differences in the subcortical projections to the LPrm and LPl remain unclear. Therefore, in this study, we used Fluorogold (FG), a retrograde neuronal tracer, to compare the source of subcortical inputs projecting axonal fibers to the LPrm and LPl.

## 2 Materials and methods

### 2.1 Animals

All animals were bred under a normal 12-h light/dark schedule and fed *ad libitum* in accordance with the National Institutes of Health Guidelines for Animal Research. All experiments were approved by the Institutional Animal Care Committee of Kurume University. Every effort was made to minimize suffering and reduce the number of animals used in this study.

### 2.2 Injection of neuronal tracers and fixation of brain

Six adult male Wistar rats (250–350 g; Japan SLC, Hamamatsu, Japan) were used in this study. Rats were anesthetized with mixed anesthesia (0.36 mg/kg medetomidine, 4.8 mg/kg midazolam, and 6.0 mg/kg butorphanol) via intraperitoneal injection and placed in a stereotaxic instrument (SR-6 M-HT; NARISIGE, Tokyo, Japan).

For retrograde labeling, rats were injected with 2.5% (w/v) FG (H22845; Thermo Fisher Scientific, Waltham, MA, United States) in 0.9% (w/v) sodium chloride iontophoretically with an Iontophoresis Pump (BAB-501; Kation Scientific, Kunfeherto, Hungary). This involved applying positive current pulses (7 s long at 7-s intervals) of 2 μA for 30 min with a glass micropipette (30–50 μm tip diameter) to the LPl (4.0 mm posterior to bregma, 2.8 mm lateral to midline, and 3.9 mm deep to brain surface) or LPrm (4.0 mm to bregma, 1.9 mm to midline, and 4.2 mm to surface).

After FG injection, rats were allowed to survive for 4 days. Subsequently, rats that received FG were deeply anesthetized using a mixture of medetomidine (0.45 mg/kg), midazolam (6.0 mg/kg), and butorphanol (7.5 mg/kg). They were then transcardially perfused with 300 mL of phosphate-buffered saline (PBS) [10 mM sodium phosphate, pH 7.4, and 0.9% (w/v) saline], followed by 300 mL fixative [0.1 M sodium phosphate buffer (PB) (pH 7.4) containing 4% (w/v) paraformaldehyde]. After perfusion, the brains were extracted and postfixed with the same fixative at room temperature for 4 h. The brains were then cryoprotected in 30% (w/v) sucrose in 0.1 M PB. Finally, the brains were coronally cut into 50 μm-thick sections with a cryomicrotome (CM1950; Leica, Wetzlar, Germany), and the sections were collected in PBS.

### 2.3 Immunoperoxidase staining

The subsequent incubations were performed at room temperature. Sections obtained from FG-injected rats were incubated overnight with a concentration of 2 μg/mL rabbit anti-FG antibody (AB153-I; Merck, Darmstadt, Germany) in PBS containing 0.3% (v/v) Triton X-100 (35501–02; Nacalai Tesque, Kyoto, Japan) and 1% albumin from bovine serum (01863–77; Nacalai) (PBS-XB). After the primary antibody reaction, the sections were washed with PBS for 10 min at two times and incubated with 1/200 diluted biotinylated donkey antibody against rabbit immunoglobulin G (711–065-152; Jackson ImmunoResearch, West Grove, PA) in PBS-XB for 2 h. Subsequently, the sections were washed twice for 10 min each with PBS. Thereafter, the sections were incubated with 1/100 diluted avidin-biotinylated peroxidase complex (VECTASTAIN Elite ABC-Peroxidase Kit Standard, PK-6100; Vector Laboratories, Newark, CA, United States) in PBS containing 0.3% (v/v) Triton X-100 for 1 h. Peroxidase bound to the sections was washed with PBS and then reacted with 0.02% (w/v) 3,3’-Diaminobenzidine (D006; Dojindo, Kumamoto, Japan) and 0.0005% (v/v) H_2_O_2_ in 50 mM Tris–HCl (pH 7.6) for 30–60 min. Next, the stained sections were mounted on the MAS-coated glass slides (SMAS-01; Matsunami, Osaka, Japan) or CREST-coated glass slides (SCRE-01; Matsunami), dried, dehydrated in an ethanol series, permeated with xylene, and coverslipped.

### 2.4 Observation of cell bodies labeled with neuronal tracer

Fifty-μm-thick sections obtained from each of the rats injected with FG into the LPl or LPrm were stained every 300 μm, and the number of FG-labeled somata was counted in the subcortical regions. After confirming the distribution of FG labeling, the architecture of the brain tissue was visualized using Nissl counterstaining with 0.1% (w/v) Cresyl fast violet (15947; Merck). Brain regions where tracer-labeled cell bodies were observed were identified based on their cytoarchitecture (Paxinos and Watson, 2007). When observing the SC, it was classified into the following two layers: superficial layers, which include the zonal, superficial gray (SGS), and optic layers, and deep layers, which include the intermediate gray and white, and deep gray and white layers. Representative sections containing FG-labeled cell bodies were photographed using an all-in-one microscope (BZ-X 710; Keyence, Osaka, Japan).

### 2.5 Statistical analysis

The percentage of the number of retrogradely labeled neurons from the LPl or LPrm in the number of all subcortical labeled cells was calculated. Subsequently, these percentages were compared using Student’s t-test. The threshold level of significance was set at *p < 0.05, **p < 0.01, and ***p < 0.001.

## 3 Results

### 3.1 Injection of retrograde tracer into the LP

**Figure 1:**
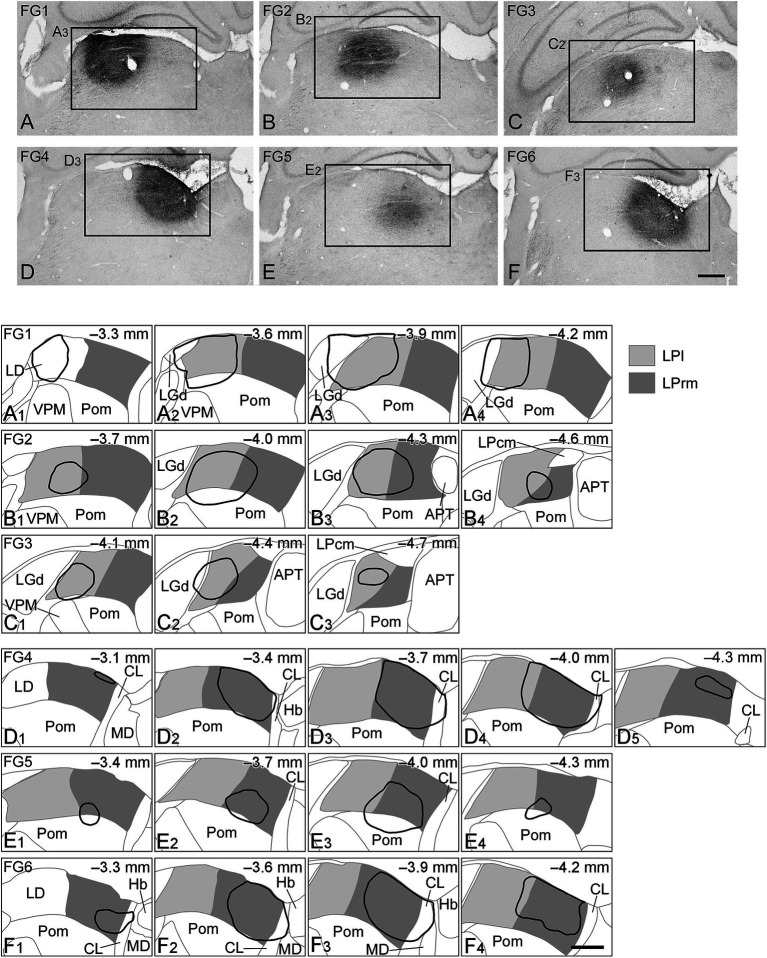
Injection sites of the retrograde tracers. Photographs of the injection sites of Fluorogold (FG) are indicated in **(A–F)**. On the drawing of the coronal plane **(A**_**1**_**–F**_**4**_**)**, the LPl or LPrm are indicated by light or dark gray, respectively, and the injection sites of FG are shown by solid black lines. In FG1 **(A**_**1**_**–A**_**4**_**)**, FG2 **(B**_**1**_**–B**_**4**_**)**, and FG3 **(C**_**1**_**–C**_**3**_**)**, FG was injected mainly in the LPl, whereas in FG4 **(D**_**1**_**–D**_**5**_**)**, FG5 **(E**_**1**_**–E**_**4**_**)**, and FG6 **(F**_**1**_**–F**_**4**_**)**, FG was injected primarily in the LPrm. The numbers in the upper right corner of **(A**_**1**_**–F**_**4**_**)** indicate the distance from Bregma. **(A**_**3**_**)**, **(B**_**2**_**)**, **(C**_**2**_**)**, **(D**_**3**_**)**, **(E**_**2**_**)**, and **(F**_**3**_**)** are part of **(A–F)**, respectively. APT, anterior pretectal nucleus; CL, centrolateral thalamic nucleus; Hb, habenular nucleus; LD, laterodorsal thalamic nucleus; LGd, dorsal lateral geniculate nucleus; LPcm, caudomedial LP; MD, mediodorsal thalamic nucleus; Pom, rostral sector of the posterior nucleus; VPM, ventral posteromedial nucleus. Scale bars in **(F)** and **(F**_**4**_**)** indicate 500 μm and apply to **(A–E)** and **(A**_**1**_**–F**_**3**_**)**, respectively.

To investigate the brain regions that send axonal fibers to the LPl and LPrm, we injected FG, a retrograde neural tracer, into each subnucleus in the LP. After injections, we visualized FGs immunohistochemically and observed their distribution. The injection sites in the rat thalamus are shown in Figure 1; in the FG1–FG3 samples, the tracer was injected into the LPl (Figures 1A–C). The injection sites were slightly extended to the dorsal lateral geniculate nucleus (LGd), rostral sector of the posterior nucleus (Pom), laterodorsal thalamic nucleus, optic tract, and LPrm adjacent to the LPl (Figures 1A_1_–C_3_). In the remaining FG4–FG6 samples, FG was injected mainly into the LPrm (Figures 1D–F); the tracer was also spread to the centrolateral thalamic nucleus (CL), Pom, and LPl surrounding the LPrm (Figures 1D_1_–F_4_).

**Figure 2:**
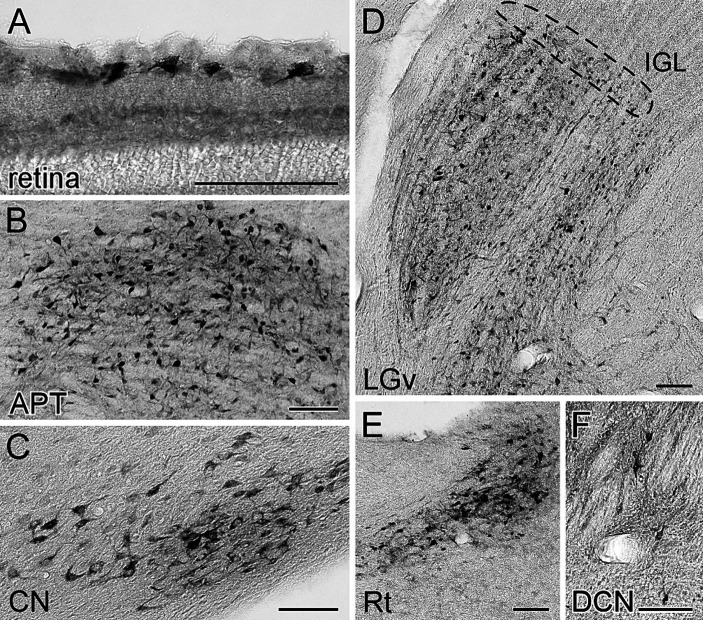
Visualization of the retrogradely labeled neurons from the LP with FG. The labeled neurons with FG were visualized by immunoperoxidase staining with anti-FG antibody. The somata of retrogradely labeled neurons from the LP were observed in the retina **(A)**, anterior pretectal nucleus **(B)**, cerebellar nucleus **(C)**, ventral lateral geniculate nucleus **(D)**, thalamic reticular nucleus **(E)**, and dorsal column nucleus **(F)**. The area indicated by the dotted line in **(D)** corresponds to the intergeniculate leaflet. Panels **(C)** and **(E)** were the sections of case FG1, while the others were derived from case FG4. APT, anterior pretectal nucleus; CN, cerebellar nucleus; DCN, dorsal column nuclei; FG, fluorogold; IGL, intergeniculate leaflet; LGv, ventral lateral geniculate nucleus; Rt, reticular thalamic nucleus. All scale bars indicate 100 μm.

Cell bodies of retrogradely labeled subcortical neurons were distributed within the retina, diencephalon, cerebellum, and brain stem (Figure 2). In the retina, superficial ganglion cells were labeled (Figure 2A), and within the thalamus, except at the injection site, labeling was observed in the thalamic reticular nucleus (Rt) (Figure 2E), the ventral lateral geniculate nucleus (LGv), and the intergeniculate leaflet (IGL) located dorsomedially to the LGv (Figure 2D). Labeled somata were also observed in the brainstem and cerebellum, including the anterior pretectal nucleus (APT) and other pretectal regions (Figure 2B), cerebellar nuclei (Figure 2C), and dorsal column nuclei (Figure 2F).

### 3.2 Retrograde labeling from the LPrm

**Figure 3:**
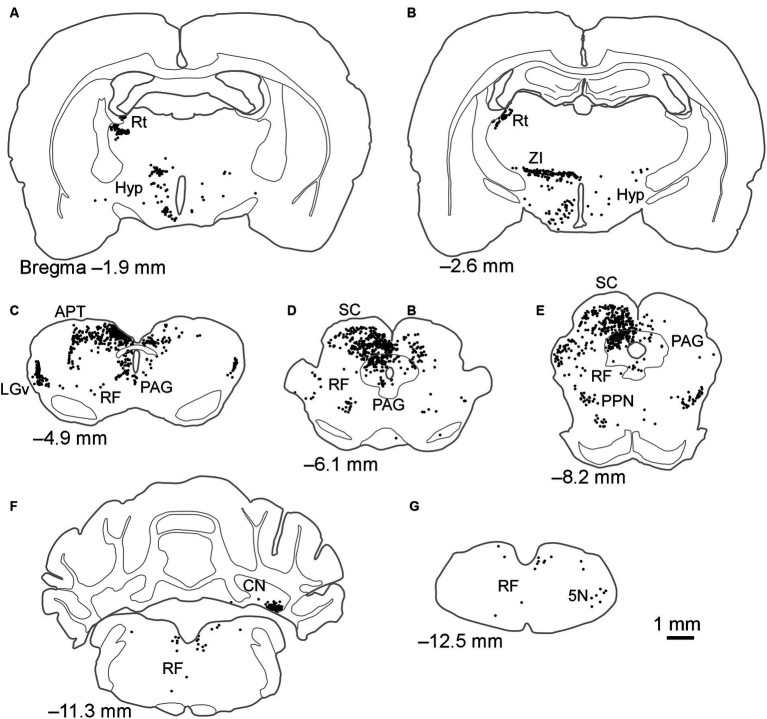
Schematics of the distributions of retrogradely labeled neurons after injection of FG into the LPrm. Dots in **(A–G)** indicate the locations of labeled cell bodies in case FG6. The left side of the drawing is ipsilateral to the FG injection site. 5 N, trigeminal nucleus; APT, anterior pretectal nucleus; CN, cerebellar nucleus; Hyp, hypothalamus; LGv, ventral lateral geniculate nucleus; PAG, periaqueductal gray; PPN, pedunculopontine tegmental nucleus; RF, reticular formation; Rt, reticular thalamic nucleus; SC, superior colliculus; ZI, zona incerta.

When FG was injected into the LPrm, retrogradely labeled neurons were found in the retina and over a wide area from the diencephalon to the medulla oblongata (Table 1; Figure 3). The retinal inputs to the LPrm were bilateral, yet a greater number of labels was distributed in the contralateral eyeball to the injection site. Within the diencephalon, cell bodies were distributed in the Rt, zona incerta (ZI), hypothalamus, LGv, and IGL (Figures 3A–C; Table 1). Of these regions, the Rt was labeled only on the injected side, while the others showed bilateral labeling, although more labeled neurons were distributed on the injected side. In the midbrain, particularly large numbers of cell bodies were labeled in the deep layers of the SC (SC-d) and periaqueductal gray (PAG) ipsilateral to the injection site. The labeling distribution within these regions was not homogeneous, with the SC-d showing more cell bodies in the medial regions than in the lateral regions and the PAG exhibiting a particularly prominent distribution in the dorsolateral region (Figures 3D,E). In the SC-s, most of the labeling was found in the optic layer, with a few labels in the SGS ipsilateral to the injection site (FG4, 85 cells; FG6, 3 cells). Furthermore, retrogradely labeled neurons were observed in the reticular formation (RF), inferior colliculus, pedunculopontine tegmental nucleus, substantia nigra, and pretectal area, including the APT (Figures 3C–E; Table 1). In all regions, more cell bodies were labeled on the injected side. In the cerebellar nuclei, cell bodies were clustered contralaterally, particularly in the ventromedial portion of the lateral nucleus (Figure 3F). In the medulla oblongata, labeling cells were found contralaterally in the trigeminal nucleus (Figure 3G).

**Table 1:** Number of retrogradely labeled cells after injections into the LP.

	Area	Injection to LPl			Injection to LPrm		
		FG1	FG2	FG3	FG4	FG5	FG6
Ipsi	Rt	379	173	101	218	100	359
	LGv	416	353	135	784	328	661
	IGL	41	48	17	119	45	103
	ZI	152	64	2	810	251	808
	Hyp	79	51	1	966	83	770
	APT	696	380	130	206	209	254
	OPT	114	53	30	27	20	86
	SN	1	10	0	78	28	96
	SC-s	76	115	118	192	14	103
	SC-d	418	170	26	3,173	308	1,634
	IC	6	4	1	351	88	99
	PAG	265	48	5	1726	346	1,514
	RF	36	20	1	353	121	624
	Other	255	222	41	700	301	634
	Retina	97	3	22	29	0	6
	Ipsi total	3,031	1714	630	9,732	2,242	7,751
Contra	LGv	104	18	7	171	2	133
	IGL	80	32	0	93	13	37
	ZI	0	0	0	132	5	66
	Hyp	22	10	0	312	6	221
	SC-s	1	0	0	7	1	5
	SC-d	27	12	1	576	45	336
	PAG	88	18	0	720	69	600
	RF	28	3	2	237	40	295
	CN	30	0	0	126	28	183
	5 N	2	3	0	17	23	34
	DCN	5	6	0	17	77	70
	Other	167	41	2	267	33	149
	Retina	800	120	451	852	9	404
	Contra total	1,354	263	463	3,527	351	2,533
	Both sides total	4,385	1977	1,093	13,259	2,593	10,284

After collected coronal serial sections were stained every 300 μm immunohistochemically, we counted the number of the labeled neurons. Data are presented as n. 5 N, trigeminal nucleus; APT, anterior pretectal nucleus; CN, cerebellar nucleus; DCN, dorsal column nuclei; Hyp, hypothalamus; IC, inferior colliculus; IGL, intergeniculate leaflet; LGv, ventral lateral geniculate nucleus; OPT, olivary pretectal nucleus; PAG, periaqueductal gray; RF, reticular formation; Rt, reticular thalamic nucleus; SC-d, deep layers of the superior colliculus; SC-s, superficial layers of the superior colliculus; SN, substantia nigra; ZI, zona incerta.

### 3.3 Retrograde labeling from the LPl

**Figure 4:**
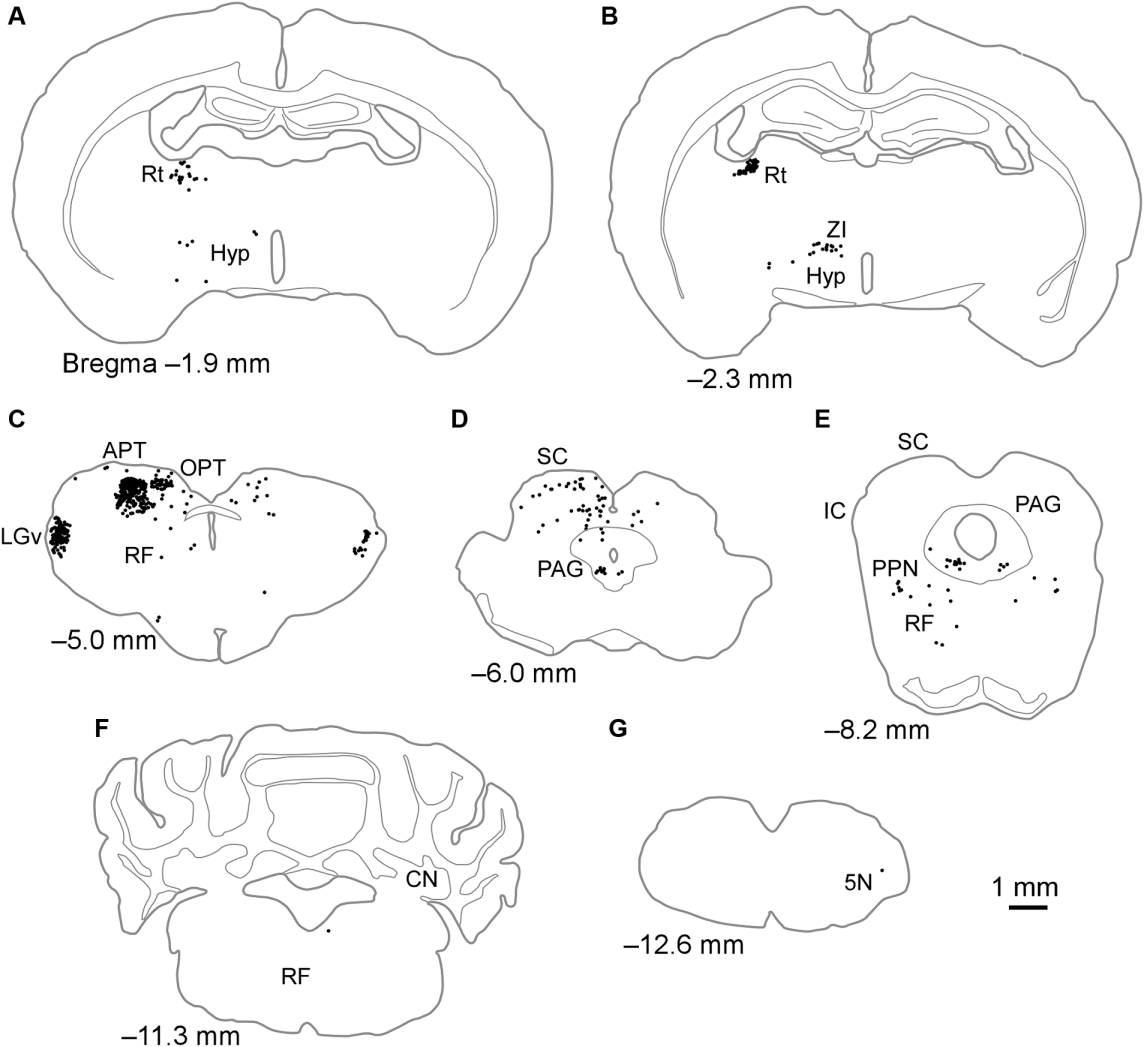
Schematics of the distributions of retrogradely labeled neurons after injection of FG into the LPl. Dots in **(A–G)** indicate the locations of labeled cell bodies in case FG1. The left side of the drawing is ipsilateral to the FG injection site. 5 N, trigeminal nucleus; APT, anterior pretectal nucleus; CN, cerebellar nucleus; Hyp, hypothalamus; IC, inferior colliculus; LGv, ventral lateral geniculate nucleus; OPT, olivary pretectal nucleus; PAG, periaqueductal gray; PPN, pedunculopontine tegmental nucleus; RF, reticular formation; Rt, reticular thalamic nucleus; SC, superior colliculus; ZI, zona incerta.

When FG was injected into the LPl, labeled neurons were mostly observed within the retina, diencephalon, and midbrain, with a few cell bodies seen in the cerebellum and medulla oblongata (Table 1; Figure 4). In the diencephalon, labeled cell bodies were distributed in the Rt, ZI, hypothalamus, LGv, and IGL, similar to injections into the LPrm (Figures 4A–C; Table 1). In the pretectal region, numerous labeled cells were observed in the dorsal part of the APT and olivary pretectal nucleus (OPT) on the same side as the injection site (Figure 4C). In other midbrain regions, the labeled neurons were observed in the RF, SC, PAG, and pedunculopontine tegmental nucleus (Figures 4C–E). Compared to injections into the LPrm, the SC-d and PAG contained fewer labeled neurons. In the SC-s, 21 labeled cells were distributed in the SGS ipsilateral to the injected site of FG1. In FG2 and FG3, all labeling was exclusively observed in the optic layer. In the cerebellum and medulla oblongata, only a few labeled cell bodies, if any, were detected (Figures 4F,G; Table 1).

### 3.4 Percentage of labeling neurons in subcortical input to the LP

**Figure 5:**
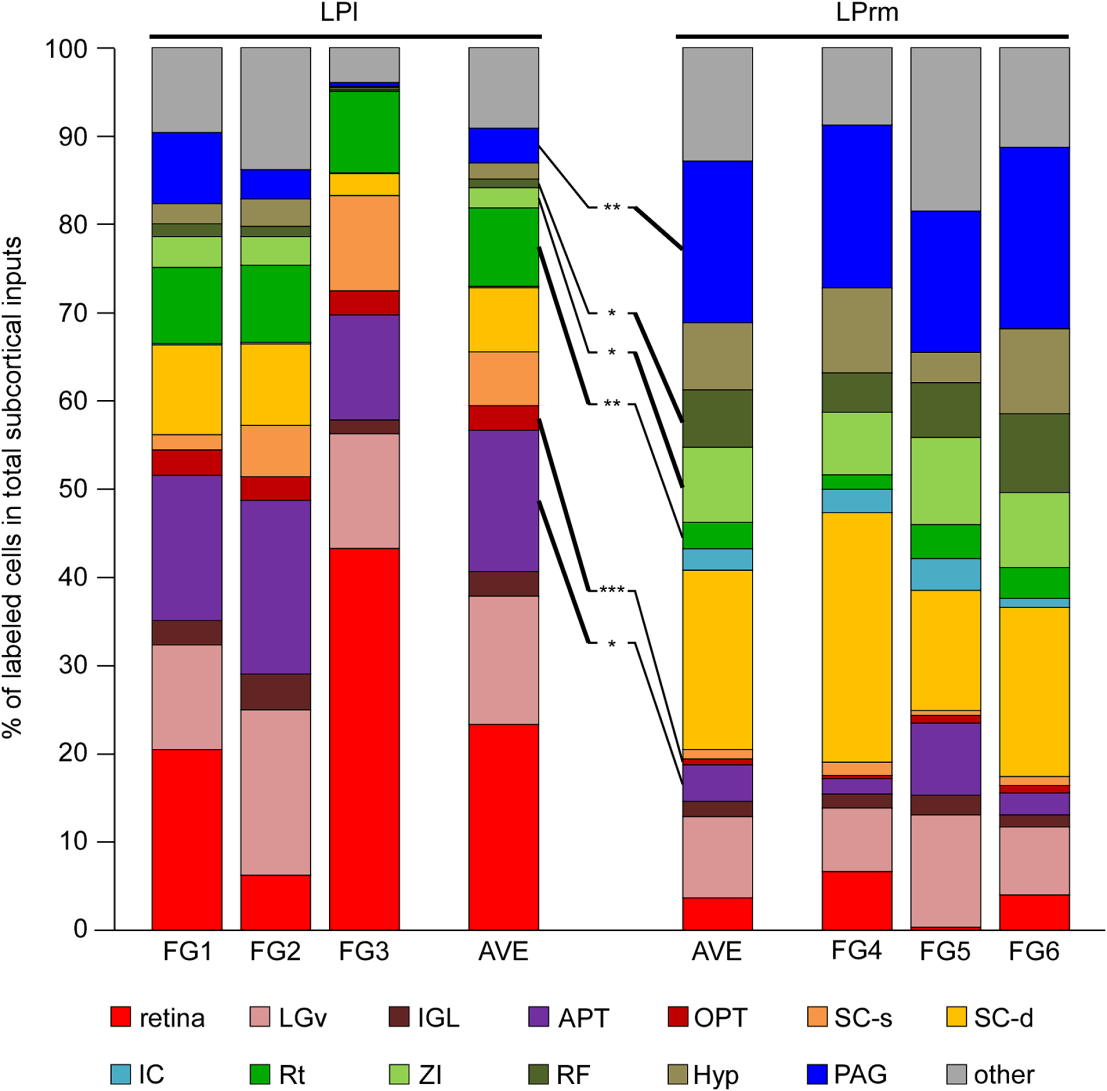
Percentage of retrogradely labeled neurons in total subcortical labeling cells. The percentages of the labeling neuron numbers in the total subcortical labeling neurons in each case (Table 2) were graphed. The values for each of the three cases of FG1–3 and FG4–6 were averaged, respectively, and compared with each other. The Rt, OPT, and APT contained predominantly higher percentages of labeled cells when injected into the LPl than when injected into the LPrm (indicated by bold lines). In contrast, the ZI, RF, and PAG were the areas with predominantly higher percentages of retrograde labeling from the LPrm. APT, anterior pretectal nucleus; Hyp, hypothalamus; IC, inferior colliculus; IGL, intergeniculate leaflet; LGv, ventral lateral geniculate nucleus; OPT, olivary pretectal nucleus; PAG, periaqueductal gray; RF, reticular formation; Rt, reticular thalamic nucleus; SC-d, deep layers of the superior colliculus; SC-s, superficial layers of the superior colliculus; ZI, zona incerta. *p < 0.05, **p < 0.01, and ***p < 0.001 using Student’s t-test.

The number of labeled neurons when the tracer was injected into the LPl tended to be lower than when it was injected into the LPrm (Figures 3, 4). However, making an exact quantitative comparison is challenging due to the difficulty of maintaining a constant injection volume of the tracer. Therefore, in this study, the number of labeled cell bodies found in each subcortical region was calculated as a percentage of the total number of the entire subcortical labeling (Table 2). The three cases where the FG was injected into the LPrm (FG4–6) showed similar distribution patterns of labeled cell bodies (Figure 5). Similarly, the three cases where the tracer was injected into the LPl (FG1–3) also demonstrated the same retrograde labeling pattern.

**Table 2:** Percentage of retrogradely labeled cells in the total subcortical labeling.

Area	Injection to LPl			Injection to LPrm		
	FG1	FG2	FG3	FG4	FG5	FG6
Retina	20.5	6.2	43.3	6.6	0.3	4.0
LGv	11.9	18.8	13.0	7.2	12.7	7.7
IGL	2.8	4.0	1.6	1.6	2.2	1.4
APT	16.5	19.7	11.9	1.7	8.2	2.5
OPT	2.9	2.7	2.7	0.4	0.8	0.8
SC-s	1.8	5.8	10.8	1.5	0.6	1.1
SC-d	10.1	9.2	2.5	28.3	13.6	19.2
IC	0.2	0.2	0.1	2.6	3.6	1.0
Rt	8.6	8.8	9.2	1.6	3.9	3.5
ZI	3.5	3.2	0.2	7.1	9.9	8.5
RF	1.5	1.2	0.3	4.4	6.2	8.9
Hyp	2.3	3.1	0.1	9.6	3.4	9.6
PAG	8.1	3.3	0.5	18.4	16.0	20.6
other	9.6	13.8	3.9	8.8	18.5	11.3

The percentage of the number of labeled cell bodies in each subcortical region (both sides) in the total number of labeled subcortical neurons was calculated. Data are presented as percentages. APT, anterior pretectal nucleus; Hyp, hypothalamus; IC, inferior colliculus; IGL, intergeniculate leaflet; LGv, ventral lateral geniculate nucleus; OPT, olivary pretectal nucleus; PAG, periaqueductal gray; RF, reticular formation; Rt, reticular thalamic nucleus; SC-d, deep layers of the superior colliculus; SC-s, superficial layers of the superior colliculus; ZI, zona incerta.

Averaging the three cases injected into the LPl, cell bodies labeled within the retina, LGv, and APT accounted for 53.8% of the total subcortical labeling (Figure 5). Averaging the injection results into the LPrm, labeled somata contained in the PAG and SC-d accounted for 38.7% of the total. Comparing the LPl and LPrm results, the predominant areas with a higher percentage of cell bodies labeled retrogradely from the LPl were the APT, OPT, and Rt, whereas the LPrm had a predominance of cell bodies labeled within the ZI, RF, and PAG.

## 4 Discussion

In this study, we determined the differences in subcortical inputs to the LPrm and LPl via retrograde tracer injections. In samples injected into the LPl, labeled neurons were abundant in the diencephalon and midbrain, with fewer somata in the pontine, cerebellum, and medulla oblongata. When the number of labeled cell bodies was calculated as a percentage of the total number of labeled neurons in the entire subcortex, over half were distributed in the retina, LGv, and APT. However, when the retrograde tracer was injected into the LPrm, labeled cells were observed in various areas, from the diencephalon to the medulla oblongata. Notably, substantial concentrations of cell bodies were observed in the SC-d and PAG, which together accounted for 38.7% of all subcortical labeling. Comparing samples where tracers were injected into the LPl and LPrm, retrogradely labeled neurons from the LPl were significantly distributed in the APT, OPT, and Rt. Conversely, the percentage of retrogradely labeled cell bodies from the LPrm was significantly higher in the ZI, RF, and PAG.

Although the LP nucleus is a thalamic nucleus involved in visual information processing, the LPrm also receives inputs from regions involved in auditory (inferior colliculus), somatosensory (dorsal column and trigeminal nuclei), and motor (deep cerebellar nuclei) functions. Previous studies have shown that the inferior colliculus, deep cerebellar nuclei, and dorsal column nuclei send axons to the LP nucleus but have not identified which subnuclei of the LP nucleus are innervated by axon fibers (Ledoux et al., 1987; Aumann et al., 1996; Ruigrok et al., 2015). Both previous reports and this study suggest that the LPrm receives a wide range of information outside the visual system when involved in visual information processing in the cortex.

After FG injection, the SC-d were among the sites with numerous labeled cell bodies. When comparing the number of labeled cells and their percentage of total subcortical input, there was a tendency for greater input to the LPrm compared to the LPl from the SC-d, although the differences were not significant (Tables 1, 2; Figure 5). This projection pattern from the SC-d to the LP aligns with previous reports (Scholl et al., 2021; Leow et al., 2022), and the SC-d is known to receive not only visual information but also auditory and somatosensory information (Sefton et al., 2015), suggesting that the LPrm receives more multisensory input than the LPl. Additionally, we showed that the labeling was more prevalent in the medial part than in the lateral part of the SC-d. The medial parts of the SC, which map the upper half of the visual field—areas where rodents frequently detect predators—have been anatomically and physiologically linked to defense responses (Comoli et al., 2012; Wei et al., 2015). These previous and present experiments suggest that the LPrm not only relays visual information but also preferentially conveys specific information that is particularly threatening to itself in the visual field.

When FG was injected into the LPrm, several labeled cell bodies were observed in the PAG, with a particular concentration of labeling around the dorsolateral portion of the PAG. Previous studies have identified axons from the PAG to the LP (Krout and Loewy, 2000), supporting these results. In the PAG, the dorsal lateral portion has been reported to receive auditory, hypothalamic, and prefrontal cortical inputs, in addition to visual inputs (Benzinger and Massopust, 1983; Newman et al., 1989; Rhoades et al., 1989; Semenenko and Lumb, 1992; Floyd et al., 2000, 2001; Gabbott et al., 2005; Goto et al., 2005; Motta et al., 2009). Additionally, the dorsolateral PAG has been implicated in triggering active defensive behavioral responses, with studies showing increased FOS expression in this region following exposure to threats such as cat odor (Dielenberg et al., 2001; Keay and Bandler, 2001; Motta et al., 2009; Dampney et al., 2013). These results, along with previous research, suggest that the LPrm likely receives multimodal sensory information and plays a role in defense responses, mediated through the PAG. Furthermore, the present study shows that these PAG-derived inputs account for a large proportion (18%) (Table 2; Figure 5) of the total subcortical inputs to the LPrm.

Whereas the LPrm receives projections from multimodal regions such as the SC-d and PAG, a large portion of the regions retrogradely labeled from the LPl were predominantly visual areas. The retina projects strongly to the LGv, IGL, OPT, and the SC-s (Yamadori, 1977; Young and Lund, 1998). The number of labeled cell bodies in these regions and the retina accounted for 49.5% of the total subcortical input after LPl injection of FG, compared to 16.3% after LPrm injection. In the pretectal region, retrogradely labeled neurons from the LPl were particularly observed in the APT in addition to the OPT. It has been reported that neurons in the OPT project to the LP in rats and to the pulvinar in monkeys and cats (Benevento and Standage, 1983; Weber et al., 1986; Klooster et al., 1995; Schmidt et al., 2001). The APT has also been reported to input to the LP in rats (Cadusseau and Roger, 1991; Leow et al., 2022), aligning with our findings. The OPT nucleus is known to receive direct retinal inputs and project to areas associated with the pupillary reflex (Klooster et al., 1995; Young and Lund, 1998). Although no reports exist on retinal inputs to the APT, it has been reported that it receives inputs from visual-associated regions such as the LGv, SC-s, and the visual cortex (Foster et al., 1989). Our results indicate that the LPl receives a higher proportion of visual information than the LPrm and is more prominently involved in visual-related functions.

Previous studies have reported differences in the corticothalamic projections to the LPl and LPrm. The primary and secondary visual cortices have a stronger projection to the LPl, while the LPrm receives projections from the rostral cortex, including the anterior cingulate and orbital cortices (Bennett et al., 2019). It has also been reported that projections from the visual cortex to the LP nucleus are primarily attributed to layer 6, whereas projections to the LPrm have more fibers originating from layer 5 (the main output layer of the cortex) compared to the LPl (Scholl et al., 2021). Furthermore, a previous study on cortical projections from the thalamic LP nucleus reported that neurons in the LPrm widely project to the visual and other cortices, whereas those in the LPl send more axons to the visual cortex than those in the LPrm (Nakamura et al., 2015). These reports suggest that the LPl plays a key role in visual function, while the LPrm participates in multimodal information processing. The anatomical differences observed in the subcortical neural circuits in this study align with previous studies on thalamocortical circuits.

This study shows that the LPl receives more subcortical projections from visual-related areas, including the retina, LGv, and OPT, while the LPrm receives more nerve fibers from areas associated with multimodal sensory and biological defense responses, such as the PAG, SC-d, ZI, and RF. Among the sites where several cell bodies were labeled in this study, LGv, IGL, APT, Rt, and ZI have been reported as brain regions that inhibit thalamic nuclei (Halassa and Acsády, 2016; Langel et al., 2018; Fratzl et al., 2021; Sabbagh et al., 2021). Since no inhibitory neurons are present in the rat dorsal thalamus, except for some thalamic nuclei, it is the prethalamus and subcortical regions outside the thalamus that inhibit the dorsal thalamic neurons. The Rt, which is a component of the prethalamus, is known to inhibit all thalamic nuclei, whereas other regions selectively project to some thalamic nuclei (Kolmac et al., 2000; Halassa and Acsády, 2016). Inhibitory innervations to the LP nucleus are reported to be from the LGv, ZI, and APT (Power et al., 1999; Kolmac et al., 2000; Moore et al., 2000). Furthermore, this study revealed a tendency for projections from the APT and ZI to the LPl and LPrm, respectively. This differential excitatory and inhibitory subcortical input to the LPl and LPrm suggests that the two subnuclei may play different functional roles. Indeed, it has also been reported that the responsiveness of neurons differs between the LPl and LPrm (Foik et al., 2020). This study indicates that the LPl and LPrm may receive information from different subcortical locations and may play different information-processing roles in the cerebral cortex.

In this study, some of the tracers did not remain exclusively within the LP nucleus (Figure 1). Therefore, some retrogradely labeled cells may project outside of the LP nucleus. Tracer leakage into the LGd occurred in cases FG1 and FG3. The LGd receives retinal input, and tectogeniculate projections from the SGS are confined to the caudal/dorsolateral shell region in the LGd (Mackay-Sim et al., 1983; Reese, 1988; Bickford et al., 2015). While a high number of labeled retinal ganglion cells were observed in both cases FG1 and FG3, the SGS had minimal labeling in FG1 and none in FG3. Thus, it is likely that the tracer leaked into the rostromedial region but did not reach the shell region of the LGd. In case FG1, the injected tracers were also observed on the optic tract; therefore, it is possible that some of the retinal ganglion cells were labeled by tracers taken up from passage fibers in the optic tract. However, in other cases, FG was injected into sites distant from the optic tract, yet labeling was observed in the retina (Table 1; Figure 5). A previous study that labeled retinal projections anterogradely found stronger labeling in the dorsal part of the LP nucleus (Yamadori, 1977; Noseda et al., 2010). Therefore, most of the retinal ganglion cells labeled in this study appear to have been labeled from the dorsal portion of the LP nucleus, and in cases FG1 and FG3, some retinal neurons may have been labeled from the LGd. Furthermore, the lack of retinal labeling in case FG5 may be due to the injection mainly into the ventral part of the LP.

Leakage of tracer to the Pom was observed in FG1, FG2, and FG5. While previous studies have reported projections from the ventral and caudal regions of the APT to the Pom (Bokor et al., 2005; Casas-Torremocha et al., 2022), retrograde labeling was more prevalent in the dorsal region of the APT in this study. The Allen Mouse Brain Connectivity Database (Allen Institute for Brain Science, 2011) confirms that anterograde tracers injected into the dorsal APT led to projections to the LPl (Experiment 264097661 and 147789031). It has also been reported that the dorsal part of the APT projects to the LP and laterodorsal thalamic nucleus (Bokor et al., 2005). In the case of FG1, tracer leakage to the laterodorsal thalamic nucleus suggests that some labeled cells may project to this nucleus. However, regardless of whether tracer leakage into the laterodorsal thalamic nucleus occurred or not, labeled APT cells accounted for a substantial proportion of the subcortical labeling in FG1–3 (Figure 5). Thus, the neurons labeled in the dorsal part of the APT in this study appear to primarily project to the LPl.

Among the findings of FG injected into the LPrm, in cases FG4 and FG6, there was tracer leakage into the CL, which is located medial to the LPrm. Previous studies have reported a projection from the SC-d to the CL (Yamasaki et al., 1986; Bickford and Hall, 1989; Masullo et al., 2019), indicating that a part of the labeled cells in cases FG4 and FG6 may also project to the CL. Although no FG leakage into the CL was observed in case FG5, we found substantial FG labeling in the SC-d (Tables 1, 2; Figure 5). Furthermore, FG labeling in the SC-d was more prevalent in the medial portions than in the lateral portions, whereas previous studies injecting retrograde tracers into the CL found labeling throughout the SC-d, not confined to only the medial or lateral part (Yamasaki et al., 1986; Bickford and Hall, 1989). Additionally, when an anterograde tracer was injected into the medial part of the SC-d, labeling in the LPrm was particularly strong (the Allen Mouse Brain Connectivity Database, Experiment 305025440 and 183376269) (Allen Institute for Brain Science, 2011). In experiment 183376269, projection fibers from the medial SC-d were mainly directed to the laterodorsal thalamic nucleus; however, labeling fibers were also distributed in the LPrm. In contrast, in experiment 305025440, where an anterograde tracer was injected into both the medial SC-s and SC-d, the main distribution site of labeled fibers was the LPcm; however, labeling was also observed in the LPrm. The labeling in the LPcm is consistent with that of previous studies, which reported that input mainly from the optic layer of the SC-s projects to the LPcm (Sugita et al., 1983; Takahashi, 1985). Therefore, based on these previous reports, the tendency in this study toward more labeling medially in the SC-d may be largely due to the projection from the SC-d to the LPrm.

This study had some limitations. First, there was difficulty in constant tracer injection and quantitative comparisons. We aimed to compare each case of injection by calculating the percentage of total subcortical input. In each case, FG1–3 and FG4–6 exhibited similar patterns, although there were differences in tracer injection sites and leakage (Figures 1, 5). This suggests that the labeling patterns obtained in this study are primarily influenced by labeling from the main injection sites, LPl and LPrm, rather than the presence or absence of tracer leakage to the surrounding LP nucleus. Second, this study focused on subcortical inputs to the thalamic nucleus but did not consider the functional properties of subcortical input fibers or cortical projections. Inhibitory projections from the APT to the LP nucleus and other thalamic nuclei have been reported in rats (Bokor et al., 2005; Leow et al., 2022), while excitatory and inhibitory projections from the pretectum to the pulvinar and LGd, respectively, have been observed in cats (Cucchiaro et al., 1991; Schmidt et al., 2001; Wang et al., 2002; Baldauf et al., 2005). The Allen Mouse Brain *in situ* hybridization database (https://mouse.brain-map.org/) has confirmed the expression of VGluT2 mRNA, a marker for excitatory cells, in the mouse APT nucleus (experiment 73818754). Therefore, further analysis using other techniques is needed to confirm whether the projection fibers to the LP nucleus are excitatory or inhibitory. Additionally, if we could analyze projections to the LP nucleus from all brain regions, including both cortical and subcortical inputs, we would be able to better clarify the influence of the LP nucleus on information processing in the brain.

In summary, our findings reveal distinct subcortical input patterns to the LPl and LPrm components of the thalamic LP nucleus. The LPrm receives strong inputs from the SC-d and PAG, along with diverse information spanning from the diencephalon to the medulla oblongata. Conversely, the majority of the projections to the LPl originate from visually relevant regions such as the retina, LGv, IGL, APT, OPT, and SC-s. These results highlight the distinct functional roles of the LPrm and LPl in visual processing. Although both the LPrm and LPl are strongly involved in visual cortical processing, this study highlights that the LPrm takes part in multisensory integration and defensive responses, whereas the LPl has a more specific role in the visual system.

## Data availability statement

The raw data supporting the conclusions of this article will be made available by the authors, without undue reservation.

## Ethics statement

The animal study was approved by the Institutional Animal Care Committee of Kurume University. The study was conducted in accordance with the local legislation and institutional requirements.

## Author contributions

HN: Writing – review & editing, Writing – original draft, Methodology, Investigation, Funding acquisition, Conceptualization. KO: Writing – review & editing, Methodology.
